# Epilepsy Associates with Decreased HIF-1α/STAT5b Signaling in Glioblastoma

**DOI:** 10.3390/cancers11010041

**Published:** 2019-01-04

**Authors:** Sharon Berendsen, Wim G. M. Spliet, Marjolein Geurts, Wim Van Hecke, Tatjana Seute, Tom J. Snijders, Vincent Bours, Erica H. Bell, Arnab Chakravarti, Pierre A. Robe

**Affiliations:** 1 Department of Neurology and Neurosurgery, Brain Center Rudolf Magnus, University Medical Center of Utrecht, Heidelberglaan 100, 3584 CX Utrecht, The Netherlands; s.berendsen-2@umcutrecht.nl (S.B.); M.Geurts-2@umcutrecht.nl (M.G.); t.seute@umcutrecht.nl (T.S.); T.J.Snijders@umcutrecht.nl (T.J.S.); 2 Department of Pathology, University Medical Center of Utrecht, Heidelberglaan 100, 3584 CX Utrecht, The Netherlands; W.G.M.Spliet@umcutrecht.nl (W.G.M.S.); w.vanhecke@umcutrecht.nl (W.V.H.); 3 Department of Human Genetics, GIGA Research Center, University of Liège, Avenue de l Hôpital, 1, 4000 Liège, Belgium; vbours@uliege.be; 4 Department of Radiation Oncology, The Ohio State University Comprehensive Cancer Center—Arthur G. James Cancer Hospital, The Ohio State University, West 10th Avenue, Columbus, OH 43210, USA; Erica.Bell@osumc.edu (E.H.B.); Arnab.Chakravarti@osumc.edu (A.C.)

**Keywords:** glioblastoma, epilepsy, translational research, GSEA, tissue microarrays, hypoxia, HIF-1α, STAT5b, mesenchymal transformation

## Abstract

Epilepsy at presentation is an independent favorable prognostic factor in glioblastoma (GBM). In this study, we analyze the oncologic signaling pathways that associate with epilepsy in human GBMs, and that can underlie this prognostic effect. Following ethical approval and patient consent, fresh frozen GBM tissue was obtained from 76 patient surgeries. Hospital records were screened for the presence of seizures at presentation of the disease. mRNA and miRNA expression-based and gene set enrichment analyses were performed on these tissues, to uncover candidate oncologic pathways that associate with epilepsy. We performed qPCR experiments and immunohistochemistry on tissue microarrays containing 286 GBMs to further explore the association of these candidate pathways and of markers of mesenchymal transformation (NF-κB, CEBP-β, STAT3, STAT5b, VEGFA, SRF) with epilepsy. Gene sets involved in hypoxia/HIF-1α, STAT5, CEBP-β and epithelial-mesenchymal transformation signaling were significantly downregulated in epileptogenic GBMs. On confirmatory protein expression analyses, epileptogenic tumors were characterized by a significant downregulation of phospho-STAT5b, a target of HIF-1α. Epilepsy status did not associate with molecular subclassification or miRNA expression patterns of the tumors. Epileptogenic GBMs correlate with decreased hypoxia/ HIF-1α/STAT5b signaling compared to glioblastomas that do not present with epilepsy.

## 1. Introduction

Glioblastoma (GBM) is the most malignant primary brain tumor with a dismal prognosis. Median patient survival in those who can undergo aggressive therapy is 15–20 months from diagnosis [1]. Genetic and epigenetic (e.g., DNA methylation, histone acetylation) events condition the deregulation of key signaling pathways in glioblastoma and hence, its growth, invasion and therapeutic resistance [2]. Specific molecular characteristics provide distinct molecular glioblastoma subtypes, of which the mesenchymal subtype has been linked to a more aggressive and invasive tumor phenotype [3] and is driven by alterations in master transcription factors such as STAT3, CEBP-β and NF-κB [4,5].

The disease presents itself with epileptic seizures in 30–40% of the cases [6,7], and we previously showed that epilepsy at presentation is an independent favorable prognostic factor for overall survival in glioblastoma patients [7]. This prognostic effect could not be explained by treatment with specific anti-epileptic drugs [7,8], a smaller tumor volume at presentation, due to an earlier detection of the tumor, or IDH1 mutations [7]. The mechanisms underlying this prognostic effect in glioblastoma thus remain to be elucidated.

Glioma-associated epilepsy is (in part) mediated by tumor specific biological changes. Most studies have however been performed in low grade gliomas (LGGs), since these tumors more often present with epilepsy compared to GBMs [9].

Tumor-associated epilepsy results, at least in part, from a local neuronal excitation/inhibition disbalance. For instance, the release of D-2-hydroxyglutarate (D2HG), a substance structurally similar to glutamate, in the tumor microenvironment was associated with epilepsy [10]. NR2B, a predominantly extrasynaptic NMDA glutamate receptor, is highly phosphorylated in peritumoral mouse brain tissue, and increases Ca^2+^ influx in the cells, leading to a self-activating circle and overexcitation of neurons [11,12]. Another proposed mechanism of tumor-associated seizures is mediated by the glutamate release pathway cystine/glutamate transporter System Xc−(SXC), which expression is elevated in a subset of glioblastoma tissues, and in peritumoral tissues. Higher glutamate concentrations and lower glutamine synthetase expression in the tumors and peritumoral tissues were also linked to glioma-related seizures [13,14,15].

Some studies have also linked glioma-associated epilepsy with *IDH1* mutation [16,17,18]. We have shown that such mutations cannot solely explain the favorable prognosis of epileptogenic glioblastomas [7]. A lower expression of *OLIG2*, linked to the proneural GBM subtype [3], was also related to an increased risk of tumor-associated seizures [19]. How this could relate to the prognosis is however unknown.

The mechanisms underlying the prognostic effect of epilepsy at presentation on overall survival in glioblastoma patients have thus not yet been investigated. Therefore, in this study, we focus on the oncogenic signaling pathways that associate with epilepsy in human glioblastomas, in search for the mechanisms that underlie this prognostic effect.

## 2. Results

Fresh-frozen tissue from 76 ‘de novo’ GBM patients was included in this study. Baseline characteristics are shown in Table S1. Of these patients, 30 presented with epilepsy and 46 had different symptoms at presentation of the disease. mRNA expression analysis was performed for 73 patients, after quality control and removal of three outliers. miRNA expression analysis was performed for 72 patients, as four samples were removed due to insufficient RNA quality. Molecular classification could be assigned to 66/76 samples. TMA’s included archival fresh-frozen paraffin-embedded (FFPE) tissue from 286 consecutive GBM patients. Details on this cohort are shown in Table S2.

### 2.1. Epileptogenic GBMs Show Downregulation of HIF1a/STAT5b Signaling

**Figure 1:**
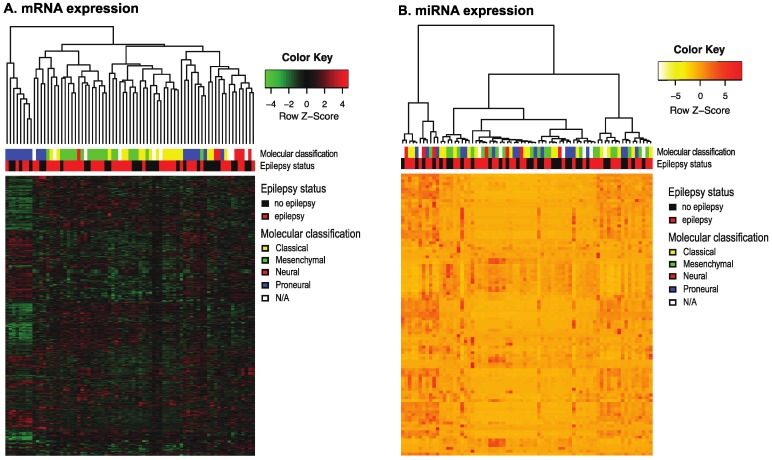
Lack of correlation between GBM-associated epilepsy and gene expression data. (**A**) Heatmap showing gene expression patterns of the 1000 RNA microarray probes with the highest standard deviation. Relative downregulation is shown as green, relative upregulation is shown as red, see color key. No differentially expressed genes were observed after correction for multiple testing (BH adjusted *p* < 0.05). (**B**) MiRNA expression patterns of the 100 probes with the highest standard deviation. 67 samples from our institute were included in this analysis. Relative miRNA downregulation is shown as white/yellow, and relative miRNA upregulation is shown as red, see color key. No differentially expressed miRNAs were observed after correction for multiple testing (Benjamini Hochberg adjusted *p* < 0.05).

None of the individual mRNAs or miRNAs reached a significant association value after correction for multiple testing (Figure 1A,B). Exploratory gene set enrichment analyses (GSEA) were performed with use of the mRNA expression data and showed significant downregulation of 218 gene sets in the epilepsy subgroup compared to GBMs that did not present with epilepsy, with use of the Broad Institute MySig (MSigDB) libraries of curated gene sets C2 collection (curated gene sets, *p* < 0.05, false discovery rate (FDR) < 0.25). The top results are shown in Table 1, and full table is available in the supplementary material (Table S3). The majority of the associated gene sets are involved in hypoxia and HIF-1α signaling. Additionally, a gene set containing STAT5 targets (*p* < 0.0001, FDR = 0.06) and gene sets involved in CEBP-β, STAT3 signaling and epithelial to mesenchymal transition (EMT) signaling (*p* < 0.05, FDR < 0.25) were downregulated in the epilepsy subgroup compared to patients without epilepsy.

GSEA with the MSigDB C3 collection containing motif gene sets showed, among others, downregulation of a gene set involved in CEBP-β signaling (*p* < 0.05, FDR < 0.25, Table 1) and SRF signaling (*p* < 0.05, FDR < 0.25). Analysis with the MSigDB C7 gene set showed downregulation of multiple gene sets containing genes responsive to LPS stimulation in macrophages (*p* < 0.05, FDR < 0.25, Table 1).

**Figure 2:**
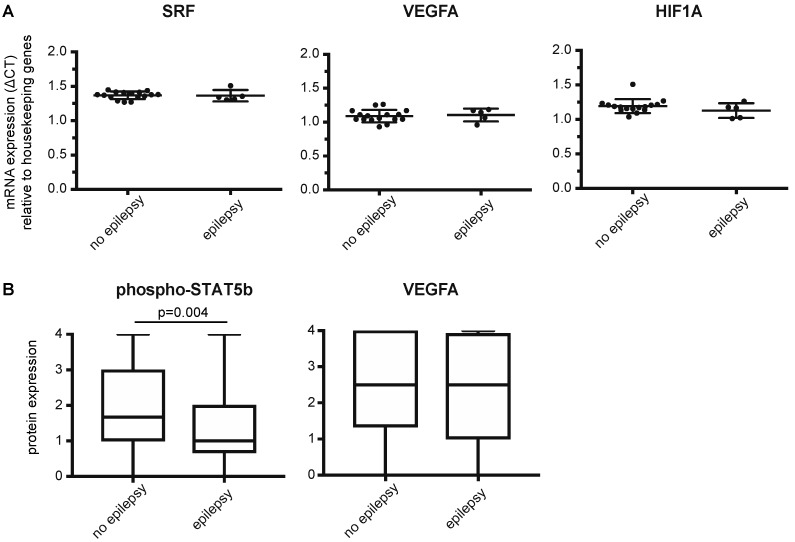
Differential activation of STAT5b between epileptogenic and non-epileptogenic GBMs. (**A**) qPCR experiments showing mRNA expression (ΔCT) of SRF, VEGFA and HIF1A in GBM patients with epilepsy (*n* = 5) and without epilepsy (*n* = 16). Graphs show mean ±SD and expression values per patient. Expression values were normalized to the average of 3 housekeeping genes (ACTB, GAPDH, GUSB). There was no significant difference in mRNA expression between epilepsy and non-epilepsy samples (independent samples *t*-test, SRF: *p* = 0.9, VEGFA: *p* = 0.76, HIF1A: *p* = 0.23). (**B**) Protein expression of phosphorylated STAT5b and VEGFA on GBM samples included on a tissue microarray. STAT5b expression was significantly lower in patients that presented with epilepsy compared to GBM patients with other presenting symptoms (*n* = 265, Mann Whitney *U* test, *p* = 0.004). VEGFA expression did not differ between the groups (*n* = 275, Mann Whitney *U* test, *p* = 0.43).

qPCR experiments in a subset of fresh-frozen GBM samples (epilepsy *n* = 5, no epilepsy *n* = 16) did not show significant differential expression of *HIF1a*, *VEGF* or *SRF* (Figure 2A). However, on a protein level, we observed a significant decrease in protein expression of nuclear phosphorylated STAT5b in the epilepsy subgroup (Mann-Whitney *U* test, *p* = 0.004, Figure 2B). The JAK/STAT5b pathway has been shown to closely correlate with hypoxia and HIF-1α signaling in multiple cancers [20,21,22,23]. VEGFA and SRF protein expression did not differ between the two groups (Figure 2B and Figure S1).

### 2.2. Epilepsy Does Not Correlate with a Mesenchymal Signature in GBMs

As described above, GSEA showed downregulation of, among others, gene sets involved in EMT, STAT3 and CEBP-β signaling in the epileptogenic GBMs. Based on these results, we further investigated the association of epileptogenic GBMs with mesenchymal transformation.

**Figure 3:**
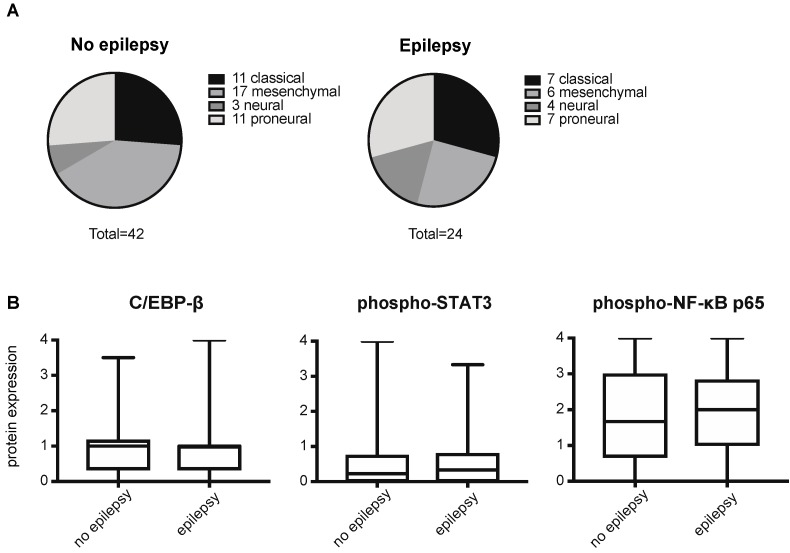
Molecular subclassification and mesenchymal activation in epileptogenic GBM. (**A**) Distribution of molecular GBM subtypes in subgroup of patients with epilepsy (*n* = 24) and without epilepsy (*n* = 42), based on mRNA expression profile. There was no significant difference in subtype distribution (Chi-square test, *p* = 0.48). (**B**) Protein expression of CEBP-β, phosphorylated STAT3 and phosphorylated NF-κB p65 on GBM tissues included on a tissue microarray. Mann Whitney *U* test did not show significant differences in protein expression between the epilepsy and non-epilepsy subgroups (CEBP-β: *n* = 266, *p* = 0.81, phosphorylated STAT3: *n* = 272, *p* = 0.68, phosphorylated NF-κB p65: *n* = 278, *p* = 0.56). Boxes represent median and quartiles, whiskers show data range.

In the subgroup of patients without epilepsy a relatively larger percentage of tumors was classified as the mesenchymal subtype compared to the epilepsy group (40% vs. 25%), but this difference did not reach statistical significance (Chi-square test, *p* = 0.48, Figure 3A). In line with these results, no significant difference was observed in protein expression of phosphorylated NF-κB p65, phosphorylated STAT3 and CEBP-β (Figure 3B), which are master transcriptional regulators of the mesenchymal gene signature in GBM.

## 3. Discussion

In our previous work, we observed that glioblastoma patients with epilepsy at the time of diagnosis have a significantly longer overall survival compared to patients that present with other symptoms. This prognostic effect was independent of anti-epileptic treatment and other important clinical characteristics [7].

Others have demonstrated that specific tumor biological effects are associated with epilepsy in glioma patients, such as neuronal excitation/inhibition disbalance and expression changes in the glutamate pathway [10,11,12,13,14,15]. Also, *IDH1* mutation was linked to the risk of tumor-associated epilepsy and seizure prognosis [16,17]. In our previous study, however, we found no correlation between epilepsy and IDH1 mutation in primary glioblastoma patients [7].

NF-κB, CEBP-β and STAT proteins, including STAT3 and −5, are transcription factors that mediate a wide range of cellular cytokine responses in physiological and disease processses [24,25,26]. Previous studies have identified STAT3 and CEBP-β as principal regulators of the mesenchymal gene expression signature in glioblastoma [4,27]. High expression of these mesenchymal markers has been associated with worse survival of GBM patients [27]. Additionally, NF-κB has been shown to drive mesenchymal transformation in glioma stem cells by induction of master transcription factors STAT3, CEBP-β and TAZ, which can subsequently contribute to resistance to radiation [5] and chemotherapy [28]. In our study, GSEA showed a downregulation of gene sets involved in epithelial to mesenchymal transformation (C2) and CEBP-β signaling (C3), suggesting a possible link between epileptogenic GBMs and molecular subclassification or mesenchymal transformation. Subsequent analyses could however not confirm this relationship. Although the epilepsy subgroup contained fewer tumors with the mesenchymal subtype, this was not a statistically significant difference. We also did not observe significant differences in protein expression of the key mesenchymal transcription factors NF-κB p65, STAT3 and CEBP-β.

Hypoxia-inducible factors, such as HIF-1α, contribute to the cell’s response to hypoxia [29]. HIF-1α is a major player in the oncologic signaling in GBM. In hypoxic conditions, a mesenchymal shift mediated by HIF-1α is induced in glioblastoma cells [30,31]. Several of the key regulators of transcription involved in mesenchymal transformation (e.g., NF-κB, CEBP-β) upregulate under hypoxic conditions in cancer cells [27,32], and hypoxia activates the JAK2/STAT5b pathway in several types of cancer [20,21,22,23]. Also, crosstalk between HIF-1α and STAT3 was reported, in different tumor types, including glioblastoma [33,34]. Interest in STAT5 signaling in glioblastoma is growing, as multiple studies have shown that STAT5b drives proliferation and invasion in glioma [35,36,37,38]. This may be, in part mediated by the oncogenic EGFRvIII variant [39,40,41].

In our study, we observed downregulation of multiple gene sets involved in HIF-1α and hypoxia signaling in the epileptogenic GBMs. Additionally, STAT5 target genes seemed downregulated in this group. Interestingly, nuclear phosphorylated STAT5b protein expression was downregulated in the epileptogenic GBMs. Activated STAT5 proteins translocate to the nucleus [26], and serine 730 phosphorylation induces its intrinsic transcriptional activity [42]. We did not observe differential RNA or protein expression of HIF-1α and its target VEGFA between the epileptogenic GBMs and tumors that did not cause epilepsy. These results indicate that epilepsy in GBM patients more specifically correlates to decreased hypoxia/HIF-1α/STAT5b signaling. Interestingly, STAT5b protein expression associated with glioblastoma patient survival in our population of patients [43].

Besides its role in tumor growth and cancerogenesis, HIF-1α/STAT5b signaling could also be directly related to the epileptogenicity of the tumors, by altering the buffering and networking properties of the glial network. STAT5-null mice have indeed undetectable levels of connexin 32 [44], while connexin 43 (Cx43) mediates HIF-1α in astrocytes [45]. High expression of connexin 43 has in turn been linked to both glioma-related [46] and epilepsy due to mesiotemporal sclerosis [47], while this latter presents reduced levels of connexin 32. On the other hand, STAT5 is a negative regulator of xCT Expression and System Xc-Activity [48]. The exploration of these potential correlations is beyond the scope of the present work but represents an interesting venue for future research. With respect to tumor epileptogenicity as well, our GSEA also showed decreased expression of gene sets involved in serum response factor (SRF) signaling in the epileptogenic tumors. SRF depletion has been associated with increased seizure frequency in a mouse model [49,50]. Interestingly, SRF has also been linked to tumor invasion, proliferation, metastasis and resistance to therapy in different cancer types [51,52,53]. Its role in glioblastoma remains to be explored. In a subset of patients however, we did not observe differential SRF RNA expression between the epilepsy and non-epilepsy subgroups by qRT-PCR. Likewise, SRF protein expression was evaluated with two different antibodies on our tissue microarrays and did not associate with epileptogenicity. Hence, we could not confirm the association of SRF signaling with epileptogenic GBMs.

Our study presents some limitations. Not all results from the gene set enrichment analyses could be confirmed by other RNA or protein expression-based experiments. This discrepancy confirms the exploratory role of GSEA. The number of fresh-frozen tumor tissues available for confirmatory qRT-PCR experiments was somewhat limited. On the other hand, we included a large cohort of patients in the TMA analyses for our confirmatory protein expression analyses. Yet, we cannot rule out that intratumoral tissue heterogeneity could alter these analyses as well. Fresh-frozen tumor specimens were all collected by an experienced neuro-oncological neurosurgeon (P.R.) from the vital tumor tissue during surgery. Due to ethical reasons, we were not able to include peritumoral brain tissue from the patients. Archival FFPE tissue was used for protein expression analyses on tissue microarrays and multiple tumor tissue regions per patient were included. To minimize this bias however, a combined protein expression score was computed per patient.

## 4. Materials and Methods

### 4.1. Ethics Statement

This study was conducted following approval by the local ethical committee, and institutional review board (protocols 09-420, 16-229, 16-348).

### 4.2. Clinical data and Tumor Tissues

Following ethical approval and written patient consent, fresh frozen glioblastoma tissue was prospectively obtained from 76 patients at first surgery for their disease between 2010–2015. Hospital records were screened for the presence of seizures at diagnosis.

### 4.3. mRNA Expression Analysis

Seventy-six fresh-frozen surgical samples of de novo GBMs were prospectively collected between 2010 and 2015. RNA was extracted with the Nucleospin^®^ TriPrep (Macherey-Nagel, Düren, Germany) and the QIASymphony RNA (Qiagen, Venlo, The Netherlands) kits according to the manufacturers’ instructions. Affymetrix HG U133 plus 2.0 arrays were prepared and scanned according to the manufacturer’s protocol and as reported previously [54]. Quality control and differential gene expression analyses were performed with R (v3.2.2). Based on principal component analysis (PCA) plots, 3 outliers were removed. Robust Multi-array Average (RMA) normalization was applied. Batch correction was performed with the ‘sva’ package. Differential expression was analyzed with the ‘limma’ package and heatmaps were created with the ‘heatmap3′ package (R).

Exploratory Gene Set Enrichment Analyses (GSEA) were performed after RMA-normalization [55] and batch correction, with the Partek^®^ Genomics suite platform (v6.6) (Partek, St. Louis, MO, USA). Analyses were performed with the Broad Institute MySig libraries of curated gene sets C1—C7 version 5.0 [56], 1000 permutations and default additional parameters. An FDR threshold of 0.25 was applied as recommended [55].

### 4.4. miRNA Expression Analysis

RNA was isolated from the 76 fresh-frozen surgical samples of GBM patients with the MiRNeasy Micro Kit (Qiagen). Expression profiling of 800 miRNA probes was performed with the nCounter^®^ Human v2 miRNA Expression Assay (NanoString, Seattle, WA, USA) at The Ohio State University Nucleic Acid Core Facility. 250 ng RNA was used per sample and conditions were set according to the manufacturer’s instructions. RNA quality was insufficient for 4 samples. Data were processed with the Partek^®^ Genomics suite platform (v6.6) by geometric mean normalization, average background subtraction and normalization to housekeeping genes. miRNA expression levels in GBMs in patients with and without seizures were analyzed with the ‘limma’ and ‘heatmap3′ package (R).

### 4.5. Class Prediction

Molecular subclassification (proneural, neural, classical, mesenchymal) was predicted by hierarchical clustering [3]. Microarray normalization, data filtering and analysis of inter-array homogeneity were performed as reported previously [3,57]. Affymetrix HG U133 plus 2.0 probesets were matched to 840 genes originally published for the classification of GBMs (http://tcga-data.nci.nih.gov/docs/publications/gbm_exp/). Relative gene expression values were calculated. Genes were then excluded for a median absolute deviation below 0.5 [3]. After filtering, 768 genes were used for the class prediction. The hierarchical clustering of samples was performed with cluster 3 software [58] with the agglomerative average linkage for the structure and 1 minus the Pearson’s correlation for the distance metric [3].

### 4.6. qPCR Analyses

RNA for qPCR was available from the RNA extraction for miRNA expression analysis for 5 GBM patients with epilepsy and 16 patients without epilepsy. RNA quality was checked by measuring of A260/280 and A260/230 values with Nanodrop. qPCR expression analyses were performed for *HIF1A*, *SRF*, *VEGFA*. Expression values were normalized to the average of 3 housekeeping genes (*ACTB*, *GAPDH*, *GUSB*).

### 4.7. Tissue Microarrays and Immunohistochemistry

Archival FFPE GBM tumor tissues from a consecutive cohort of 286 patients treated in the UMCU between 2009 and 2013 were included on tissue microarrays (TMA’s) as described previously [7]. Immunohistochemistry was performed, as described previously [7], with antibodies against STAT5b (phospho S731, Rabbit polyclonal, Abcam, Cambridge, UK), VEGF (Rabbit polyclonal, ThermoScientific, Waltham, MA USA), anti-NF-κB p65 (phospho S276, Rabbit polyclonal, Abcam); anti-STAT3 (phospho Y705) (Rabbit monoclonal, Cell Signaling, Leiden, The Netherlands); anti-CEBP-β (Mouse monoclonal, Abcam); anti-SRF (Rabbit polyclonal, Abcam, and rabbit polyclonal, Sigma-Aldrich, St. Louis, MO, USA).

Protein expression evaluation was performed with blinding for the clinical data, and was supervised by a senior neuropathologist. The percentage of nuclear and/or cytoplasmatic staining was scored as: 0, negative; 1, 1–25% positive cells; 2, 26–50% positive cells; 3, 51–75% positive cells and 4, 76–100% positive cells. A mean expression score was computed per patient. Due to insufficient tissue quality on TMA, a variable number of tissues per staining could not be evaluated (*n* = 8–21).

### 4.8. Statistical Analyses

Expression analyses of individual mRNAs and miRNAs were performed with the ‘limma’ package in R (v3.2.2). To control for inflation of type I error by multiple testing, *p*-values were adjusted by default Benjamini-Hochberg procedure. Adjusted *p*-values < 0.05 were considered significant.

GSEA was performed with the Partek^®^ Genomics suite platform (v6.6). A recommended cutoff of *p* < 0.05 and false-discovery rate (FDR) < 0.25 was applied. All other analyses were performed with SPSS (v25, IBM, Armonk, NY, USA). A *p*-value < 0.05 was considered significant. Differential distributions of molecular subtypes across epilepsy and non-epilepsy patients were tested with a Chi-square test. Differences in RNA expression between patients with and without epilepsy were analyzed by independent samples *t*-test. Data distribution of protein expression was evaluated graphically and with a Kolmogorov-Smirnov test and differential expression was subsequently analyzed with a Mann Whitney *U* test.

## 5. Conclusions

A reduced activity of the hypoxia/HIF-1α/STAT5b signaling pathway is associated with epileptogenicity in glioblastomas. For the first time to our knowledge, these results provide biological insight in the favorable prognosis of tumor-associated epilepsy.

## Figures and Tables

**Table 1:** Gene set enrichment analysis with MSigDB collections. Gene sets are significantly downregulated in the epilepsy group compared to the patients without epilepsy. Cutoff values for significance were *p* < 0.05 and FDR < 0.25. Analyses were performed with MSigDB collections C1–C7. A subset of significant C2 results were displayed in this table. See supplementary Table S1 for full results with the C2 collection. NES: normalized enrichment score, FDR: false-discovery rate.

Gene Sets	NES	*p*-Value	FDR
C2 collection—curated gene sets			
ELVIDGE_HIF1A_TARGETS_DN	−2.355	<0.0001	<0.0001
ELVIDGE_HYPOXIA_BY_DMOG_UP	−2.283	<0.0001	0.0007
ELVIDGE_HIF1A_AND_HIF2A_TARGETS_DN	−2.301	<0.0001	0.0007
ELVIDGE_HYPOXIA_UP	−2.259	<0.0001	0.0009
LEONARD_HYPOXIA	−2.285	<0.0001	0.001
FARDIN_HYPOXIA_11	−2.198	<0.0001	0.007
PID_HIF1_TFPATHWAY	−2.149	<0.0001	0.02
GROSS_HIF1A_TARGETS_DN	−2.136	<0.0001	0.02
GROSS_HYPOXIA_VIA_ELK3_AND_HIF1A_UP	−2.116	<0.0001	0.03
C3 collection—transcription factor targets			
V$ROAZ_01	−1.69	0.03	0.11
V$SRF_01	−1.71	0.02	0.13
CCAWWNAAGG_V$SRF_Q4	−1.71	0.01	0.16
TTGCWCAAY_V$CEBPB_02	−1.73	0.004	0.18
GGGNRMNNYCAT_UNKNOWN	−1.62	0.006	0.20
KRCTCNNNNMANAGC_UNKNOWN	−1.77	0.013	0.25
C7 collection—immunologic signatures			
GSE14769_UNSTIM_VS_40MIN_LPS_BMDM_DN	−1.90	0.006	0.13
GSE37416_CTRL_VS_12H_F_TULARENSIS_LVS_NEUTROPHIL_DN	−1.90	0.006	0.15
GSE14769_UNSTIM_VS_80MIN_LPS_BMDM_DN	−1.91	0.008	0.17
GSE14769_UNSTIM_VS_60MIN_LPS_BMDM_DN	−1.91	0.006	0.21
GSE37416_CTRL_VS_3H_F_TULARENSIS_LVS_NEUTROPHIL_DN	−1.93	0.004	0.24

## Supplementary Materials

The following are available online at http://www.mdpi.com/2072-6694/11/1/41/s1, Figure S1: SRF protein expression does not correlate to GBM-associated epilepsy, Table S1: Baseline table fresh-frozen tissues, Table S2: Baseline table TMA cohort, Table S3: GSEA results MSigDB C2 collection.

## Abbreviations

ACTB	Actin β
CEBP-β	CCAAT/Enhancer Binding Protein β
D2HG	(D)-2-hydroxyglutarate
EGFRvIII	Epidermal growth factor receptor variant III
GAPDH	Glyceraldehyde 3-phosphate dehydrogenase
GUSB	β-glucuronidase
HIF-1α	Hypoxia-inducible factor-1α
IDH1	Isocitrate dehydrogenase 1
JAK	Janus kinase
LPS	Lipopolysaccharide
NF-kB	Nuclear Factor binding near the κ light chain gene in B cells
NMDA	N-methyl-D-aspartate
NR2B	N-methyl D-aspartate receptor subtype 2B
OLIG2	Oligodendrocyte transcription factor
SRF	Serum response factor
STAT3	Signal transducer of activation 3
STAT5	Signal transducer of activation 5
TAZ	Tafazzin
VEGF	Vascular endothelial growth factor
