# Supplementary material for: Epilepsy Associates with Decreased HIF-1α/STAT5b Signaling in Glioblastoma
Source: Cancers (Basel). 2019 Jan 4;11(1):41. doi: 10.3390/cancers11010041 (PMC6356242; doi:10.3390/cancers11010041)
Supplement: Supplementary file 1 [file cancers-11-00041-s001.pdf]

cancers

Supplementary Materials: Epilepsy Associates with Decreased HIF-1α/STAT5b Signaling in Glioblastoma

Sharon Berendsen, Wim G. M. Spliet, Marjolein Geurts, Wim Van Hecke, Tatjana Seute, Tom J. Snijders, Vincent Bours, Erica H. Bell, Arnab Chakravarti, and Pierre A. Robe

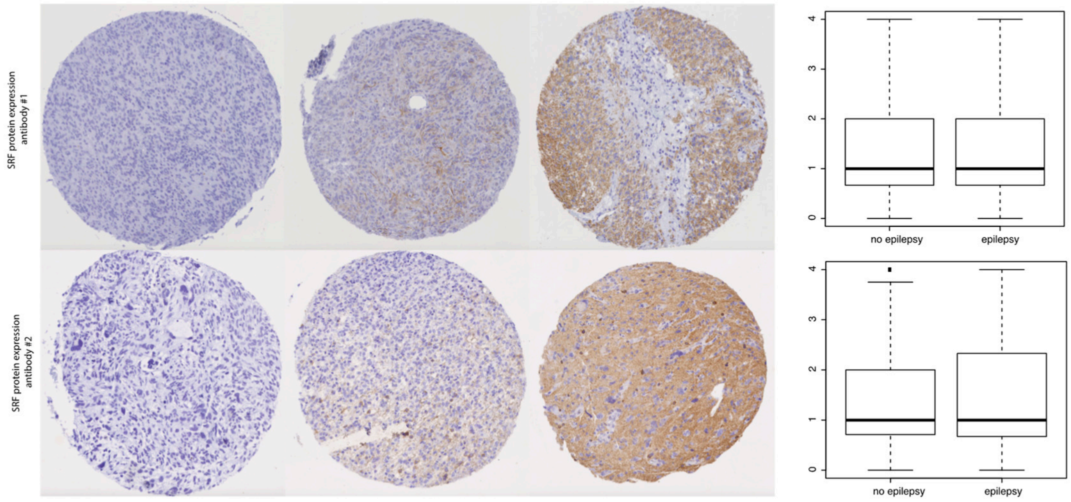

Supplementary Figure S1. SRF protein expression does not correlate to GBM-associated epilepsy. SRF protein expression on GBM tissues included on a tissue microarray. Shown are examples of GBM tissues with low, moderate and high SRF expression. Boxes represent median and quartiles, whiskers show data range. Upper panel: anti-SRF (rabbit polyclonal, Sigma, n = 297, Mann Whitney U test p = 0.63, lower panel: anti-SRF (Rabbit polyclonal, Abcam, n = 290, Mann Whitney U test p = 0.94).

Supplementary Table S1 – Baseline table fresh frozen tissues.

**Patient characteristics** ***n (%)***	**Epilepsy** *31 (40.7)*	**No Epilepsy** *45 (59.2)*
**Age** ***(mean ± SD)***	57.6 ± 10.9	63.4 ± 9.0
**Gender** ***(% male)***	71	64.4
**KPS n (%)**		
**< 70**	4 (12.9)	7 (15.6)
**≥ 70**	27 (87.1)	38 (84.4)
**Extent of surgery** ***n (%)***		
**Biopsy**	2 (6.5)*	2 (4.4)*
**Debulking**	31 (100)	45 (100)
**Post-surgical treatment** ***n (%)***		
**None**	1 (3.2)	4 (8.9)
**Monotherapy RT or TMZ**	5 (16.1)	12 (26.7)
**RT + TMZ**	25 (80.6)	29 (64.4)

Abbreviations: KPS: Karnofsky performance score; RT: radiotherapy; TMZ: temozolomide. * 4 patients received a biopsy first followed by debulking.

Supplementary Table S2 – Baseline table TMA cohort.

**Patient characteristics** ***n (%)***	**Epilepsy** *102 (35.7)*	**No Epilepsy** *184 (64.3)*	**Statistics**
**Age** ***(mean ± SD)***	57.8 ± 13.4	61.7 ± 12.0	Mann Whitney *U* test p = 0.02
**Gender** ***(% male)***	64.7	57.6	Chi-square $p = 0.24$
**KPS n (%)**			
**< 70**	21 (20.6)	50 (27.2)	Chi-square $p = 0.23$
**≥ 70**	80 (78.4)	134 (72.8)	
	Missing: 1 (1)		
**Extent of surgery** ***n (%)***			
**Biopsy**	9 (8.8)	4 (2.2)	Fisher exact $p = 0.015$
**Debulking**	93 (91.2)	180 (97.8)	
**Post-surgical treatment** ***n (%)***			
**None**	9 (8.8)	27 (14.7)	Chi-square $p = 0.067$
**Monotherapy RT or TMZ**	17 (16.7)	44 (23.9)	
**RT + TMZ**	76 (74.5)	111 (60.3)	
		Missing: 2 (1.1)	

Abbreviations: KPS: Karnofsky performance score; RT: radiotherapy; TMZ: temozolomide

Supplementary Table S3 – GSEA results MSigDB C2 collection. Gene sets downregulated in epilepsy group compared to GBM patients without epilepsy. $p < 0.05$, FDR < 0.25, NES: Normalized enrichment score.

Gene Set	NES	*p*-Value	FDR
ELVIDGE_HIF1A_TARGETS_DN	-2.35	<0.0001	<0.0001
ELVIDGE_HYPOXIA_BY_DMOG_UP	-2.28	<0.0001	0.001
ELVIDGE_HIF1A_AND_HIF2A_TARGETS_DN	-2.30	<0.0001	0.001
ELVIDGE_HYPOXIA_UP	-2.26	<0.0001	0.001
LEONARD_HYPOXIA	-2.29	<0.0001	0.001
FARDIN_HYPOXIA_11	-2.20	<0.0001	0.007
PID_HIF1_TFPATHWAY	-2.15	<0.0001	0.017
GROSS_HIF1A_TARGETS_DN	-2.14	<0.0001	0.020
GROSS_HYPOXIA_VIA_ELK3_AND_HIF1A_UP	-2.12	<0.0001	0.027
WANG_ADIPOGENIC_GENES_REPRESSED_BY_SIRT1	-2.10	0.002	0.031
MAINA_VHL_TARGETS_DN	-2.10	0.002	0.033
MENSE_HYPOXIA_UP	-2.08	0.002	0.033
AMIT_EGF_RESPONSE_40_HELA	-2.08	<0.0001	0.033
SEMENZA_HIF1_TARGETS	-2.08	<0.0001	0.035
KRIGE_AMINO_ACID_DEPRIVATION	-2.08	0.006	0.036
APRELIKOVA_BRCA1_TARGETS	-2.07	<0.0001	0.036
PRAMOONJAGO_SOX4_TARGETS_UP	-2.04	0.002	0.051
PID_FGF_PATHWAY	-2.04	<0.0001	0.055
BERENJENO_ROCK_SIGNALING_NOT_VIA_RHOA_DN	-2.03	<0.0001	0.060
HARRIS_HYPOXIA	-2.02	<0.0001	0.061
WIERENGA_STAT5A_TARGETS_GROUP2	-2.02	<0.0001	0.064
KIM_HYPOXIA	-2.00	<0.0001	0.069
PID_AVB3_OPN_PATHWAY	-2.00	<0.0001	0.069
SIMBULAN_UV_RESPONSE_NORMAL_DN	-2.00	<0.0001	0.070
VANDESLUIS_COMMD1_TARGETS_GROUP_2_UP	-2.01	0.002	0.071
ZHU_CMV_24_HR_DN	-1.99	0.002	0.076
LU_TUMOR_ANGIOGENESIS_UP	-1.99	<0.0001	0.076
NOJIMA_SFRP2_TARGETS_UP	-1.99	<0.0001	0.078
REACTOME_GLYCOLYSIS	-1.98	0.004	0.080
PICCALUGA_ANGIOIMMUNOBLASTIC_LYMPHOMA_DN	-1.98	<0.0001	0.082
TOMIDA_METASTASIS_DN	-1.94	0.006	0.097
REACTOME_REGULATION_OF_INSULIN_LIKE_GROWTH_FACTOR_IGF_ACTIVITY_BY_INSULIN_LIKE_GROWTH_FACTOR_BINDING_PROTEINS_IGFBPS	-1.94	0.002	0.097
QI_HYPOXIA	-1.96	<0.0001	0.098
BIOCARTA_HIF_PATHWAY	-1.95	0.002	0.098
LI_WILMS_TUMOR_VS_FETAL_KIDNEY_2_DN	-1.95	<0.0001	0.098

Supplementary Table S3. *Cont.*

DASU_IL6_SIGNALING_DN	-1.95	0.002	0.099
BURTON_ADIPOGENESIS_1	-1.96	0.002	0.099
CHEN_LUNG_CANCER_SURVIVAL	-1.95	0.004	0.100
BIOCARTA_GLYCOLYSIS_PATHWAY	-1.95	0.002	0.100
ABRAHAM_ALPC_VS_MULTIPLE_MYELOMA_UP	-1.96	0.002	0.100
DASU_IL6_SIGNALING_SCAR_DN	-1.94	0.006	0.100
GALINDO_IMMUNE_RESPONSE_TO_ENTEROTOXIN	-1.96	<0.0001	0.101
SASSON_FSH_RESPONSE	-1.93	0.002	0.105
NGO_MALIGNANT_GLIOMA_1P_LOH	-1.92	0.002	0.105
CHEN_LVAD_SUPPORT_OF_FAILING_HEART_UP	-1.92	0.002	0.105
ZIRN_TRETINOIN_RESPONSE_UP	-1.93	<0.0001	0.105
DELACROIX_RAR_TARGETS_DN	-1.93	<0.0001	0.106
TSAI_RESPONSE_TO_RADIATION_THERAPY	-1.93	<0.0001	0.106
JIANG_AGING_CEREBRAL_CORTEX_UP	-1.92	<0.0001	0.106
WEIGEL_OXIDATIVE_STRESS_BY_TBH_AND_H2O2	-1.92	0.004	0.106
ZHU_CMV_ALL_DN	-1.93	0.004	0.108
KAN_RESPONSE_TO_ARSENIC_TRIOXIDE	-1.92	<0.0001	0.108
PODAR_RESPONSE_TO_ADAPHOSTIN_UP	-1.92	0.002	0.109
SWEET_KRAS_TARGETS_UP	-1.91	0.006	0.109
MCDOWELL_ACUTE_LUNG_INJURY_UP	-1.91	0.002	0.110
KEEN_RESPONSE_TO_ROSIGLITAZONE_DN	-1.91	0.002	0.110
DELASERNA_TARGETS_OF_MYOD_AND_SMARCA4	-1.91	0.002	0.110
CAFFAREL_RESPONSE_TO_THC_24HR_3_UP	-1.90	0.002	0.114
FUNG_IL2_SIGNALING_2	-1.90	0.002	0.114
WACKER_HYPOXIA_TARGETS_OF_VHL	-1.90	0.008	0.115
GROSS_ELK3_TARGETS_DN	-1.90	0.008	0.116
VERRECCHIA_RESPONSE_TO_TGFB1_C3	-1.89	<0.0001	0.117
JECHLINGER_EPITHELIAL_TO_MESENCHYMAL_TRANSITION_DN	-1.89	<0.0001	0.117
PHONG_TNF_RESPONSE_VIA_P38_PARTIAL	-1.90	0.004	0.118
MOOTHA_GLYCOLYSIS	-1.89	0.004	0.118
ZAIDI_OSTEOBLAST_TRANSCRIPTION_FACTORS	-1.89	<0.0001	0.118
MACLACHLAN_BRCA1_TARGETS_DN	-1.89	0.008	0.118
GENTILE_UV_RESPONSE_CLUSTER_D4	-1.89	<0.0001	0.120
AMIT_EGF_RESPONSE_60_HELA	-1.88	0.010	0.121
OXFORD_RALA_OR_RALB_TARGETS_DN	-1.88	0.002	0.122
DALESSIO_TSA_RESPONSE	-1.88	0.012	0.122
HOLLEMAN_VINCRISTINE_RESISTANCE_B_ALL_DN	-1.88	0.006	0.122
NAGASHIMA_NRG1_SIGNALING_UP	-1.88	0.002	0.124
IYENGAR_RESPONSE_TO_ADIPOCYTE_FACTORS	-1.88	<0.0001	0.124
PHONG_TNF_TARGETS_UP	-1.88	0.010	0.125

Supplementary Table S3. *Cont.*

RODRIGUES_THYROID_CARCINOMA_DN	-1.88	<0.0001	0.126
BIOCARTA_41BB_PATHWAY	-1.86	<0.0001	0.148
HAN_JNK_SINGALING_UP	-1.85	0.004	0.149
NAGASHIMA_EGF_SIGNALING_UP	-1.86	0.006	0.149
SASSON_RESPONSE_TO_FORSKOLIN_DN	-1.85	<0.0001	0.150
MARTORIATI_MDM4_TARGETS_FETAL_LIVER_UP	-1.85	0.004	0.150
XU_HGF_TARGETS_INDUCED_BY_AKT1_6HR	-1.85	0.004	0.152
IIZUKA_LIVER_CANCER_PROGRESSION_G1_G2_DN	-1.84	0.002	0.153
ROZANOV_MMP14_TARGETS_SUBSET	-1.85	0.004	0.154
GARGALOVIC_RESPONSE_TO_OXIDIZED_PHOSPHOLIPIDS_TURQUOISE_UP	-1.84	<0.0001	0.155
GROSS_HYPOXIA_VIA_ELK3_DN	-1.84	0.002	0.156
BROWNE_HCMV_INFECTION_24HR_DN	-1.85	0.004	0.156
MAHADEVAN_RESPONSE_TO_MP470_DN	-1.84	0.018	0.156
HUMMERICH_BENIGN_SKIN_TUMOR_DN	-1.84	0.004	0.157
REACTOME_GLUCONEOGENESIS	-1.84	0.002	0.157
BASSO_HAIRY_CELL_LEUKEMIA_DN	-1.83	0.004	0.168
TIAN_TNF_SIGNALING_NOT_VIA_NFKB	-1.83	0.008	0.169
AMIT_DELAYED_EARLY_GENES	-1.83	0.010	0.169
NIELSEN_GIST_VS_SYNOVIAL_SARCOMA_UP	-1.82	0.010	0.173
CAIRO_PML_TARGETS_BOUND_BY_MYC_DN	-1.82	<0.0001	0.174
KEGG_STARCH_AND_SUCROSE_METABOLISM	-1.82	0.006	0.175
VERRECCHIA_RESPONSE_TO_TGFB1_C1	-1.82	0.012	0.176
HALMOS_CEBPA_TARGETS_DN	-1.82	0.002	0.177
SHAFFER_IRF4_MULTIPLE_MYELOMA_PROGRAM	-1.82	0.006	0.179
DANG_MYC_TARGETS_DN	-1.81	0.006	0.179
BOGNI_TREATMENT_RELATED_MYELOID_LEUKEMIA_UP	-1.81	0.008	0.180
BREUHAHN_GROWTH_FACTOR_SIGNALING_IN_LIVER_CANCER	-1.81	0.002	0.180
TRACEY_RESISTANCE_TO_IFNA2_DN	-1.82	0.004	0.180
BRACHAT_RESPONSE_TO_METHOTREXATE_DN	-1.81	0.016	0.181
KORKOLA_CHORIOCARCINOMA_UP	-1.81	0.008	0.182
TRAYNOR_RETT_SYNDROM_DN	-1.81	0.006	0.182
MARCHINI_TRABECTEDIN_RESISTANCE_DN	-1.80	0.016	0.183
MASRI_RESISTANCE_TO_TAMOXIFEN_AND_AROMATASE_INHIBITORS_DN	-1.80	0.012	0.183
TRAYNOR_RETT_SYNDROM_UP	-1.81	0.008	0.183
GNATENKO_PLATELET_SIGNATURE	-1.81	0.012	0.184
PETRETTO_CARDIAC_HYPERTROPHY	-1.81	0.010	0.184
DELASERNA_MYOD_TARGETS_DN	-1.79	0.002	0.185
REACTOME_HYALURONAN_METABOLISM	-1.79	0.018	0.185

Supplementary Table S3. *Cont.*

WANG_NEOPLASTIC_TRANSFORMATION_BY_CCND1_MYC	-1.80	0.012	0.186
OSADA_ASCL1_TARGETS_DN	-1.80	0.013	0.186
CHEN_PDGF_TARGETS	-1.79	<0.0001	0.186
PID_HIF2PATHWAY	-1.80	0.006	0.186
HOLLEMAN_PREDNISOLONE_RESISTANCE_B_ALL_DN	-1.79	0.006	0.187
REACTOME_SIGNALING_BY_ACTIVATED_POINT_MUTANTS_OF_FGFR1	-1.79	0.002	0.188
XU_HGF_SIGNALING_NOT_VIA_AKT1_48HR_UP	-1.80	0.012	0.188
DACOSTA_LOW_DOSE_UV_RESPONSE_VIA_ERCC3_XPCS_DN	-1.79	0.006	0.188
PID_AVB3_INTEGRIN_PATHWAY	-1.80	0.008	0.189
WANG_RESPONSE_TO_PACLITAXEL_VIA_MAPK8_UP	-1.80	0.010	0.189
DIRMEIER_LMP1_RESPONSE_EARLY	-1.79	0.019	0.189
TIEN_INTESTINE_PROBIOTICS_6HR_UP	-1.78	0.014	0.190
SCHAEFFER_PROSTATE_DEVELOPMENT_12HR_UP	-1.78	<0.0001	0.190
TIEN_INTESTINE_PROBIOTICS_2HR_UP	-1.79	0.016	0.190
DUNNE_TARGETS_OF_AML1_MTG8_FUSION_DN	-1.78	0.010	0.191
VERRECCHIA_EARLY_RESPONSE_TO_TGFB1	-1.78	0.006	0.191
DOANE_BREAST_CANCER_CLASSES_DN	-1.78	0.004	0.191
DAUER_STAT3_TARGETS_UP	-1.79	0.018	0.191
MOOTHA_PYR	-1.78	0.014	0.192
HERNANDEZ_MITOTIC_ARREST_BY_DOCETAXEL_2_UP	-1.78	0.002	0.192
BERENJENO_TRANSFORMED_BY_RHOA_REVERSIBLY_DN	-1.78	0.008	0.193
BURTON_ADIPOGENESIS_PEAK_AT_2HR	-1.78	0.006	0.194
MIZUKAMI_HYPOXIA_UP	-1.78	0.004	0.194
FERRARI_RESPONSE_TO_FENRETINIDE_UP	-1.77	0.014	0.196
BOYLAN_MULTIPLE_MYELOMA_C_CLUSTER_DN	-1.77	0.008	0.197
MARTORIATI_MDM4_TARGETS_NEUROEPITHELIUM_UP	-1.77	0.010	0.198
KIM_WT1_TARGETS_8HR_UP	-1.76	0.002	0.202
BIOCARTA_ARENRF2_PATHWAY	-1.77	0.008	0.203
SCIAN_INVERSED_TARGETS_OF_TP53_AND_TP73_DN	-1.77	0.011	0.203
AMIT_SERUM_RESPONSE_240_MCF10A	-1.77	0.004	0.204
BROCKE_APOPTOSIS_REVERSED_BY_IL6	-1.76	0.010	0.204
FRIDMAN_SENESCENCE_UP	-1.76	0.015	0.204
FRIDMAN_IMMORTALIZATION_DN	-1.76	0.015	0.204
DITTMER_PTHLH_TARGETS_DN	-1.76	0.002	0.205
RASHI_RESPONSE_TO_IONIZING_RADIATION_2	-1.76	0.014	0.207
STEGER_ADIPOGENESIS_DN	-1.76	0.012	0.208
BURTON_ADIPOGENESIS_9	-1.76	0.008	0.208
LABBE_TGFB1_TARGETS_UP	-1.75	0.008	0.208
VANDESLUIS_COMMD1_TARGETS_GROUP_3_UP	-1.75	0.018	0.208
GEISS_RESPONSE_TO_DSRNA_UP	-1.75	0.030	0.209

Supplementary Table S3. *Cont.*

MATTHEWS_AP1_TARGETS	-1.76	0.025	0.209
LEE_CALORIE_RESTRICTION_MUSCLE_UP	-1.76	0.018	0.209
VERRECCHIA_RESPONSE_TO_TGFB1_C4	-1.75	0.014	0.210
REACTOME_PYRUVATE_METABOLISM	-1.75	0.019	0.214
ZWANG_CLASS_2_TRANSIENTLY_INDUCED_BY_EGF	-1.74	0.004	0.216
PID_FOXO_PATHWAY	-1.74	0.002	0.216
KYNG_ENVIRONMENTAL_STRESS_RESPONSE_UP	-1.75	0.024	0.216
AGARWAL_AKT_PATHWAY_TARGETS	-1.74	0.014	0.217
AMIT_SERUM_RESPONSE_120_MCF10A	-1.74	0.008	0.217
BRUECKNER_TARGETS_OF_MIRLET7A3_DN	-1.75	0.020	0.217
AZARE_NEOPLASTIC_TRANSFORMATION_BY_STAT3_UP	-1.74	0.010	0.217
SPIELMAN_LYMPHOBLAST_EUROPEAN_VS_ASIAN_2FC_DN	-1.74	0.019	0.218
MYLLYKANGAS_AMPLIFICATION_HOT_SPOT_9	-1.74	0.014	0.220
KOBAYASHI_EGFR_SIGNALING_6HR_DN	-1.74	0.017	0.220
GERY_CEBP_TARGETS	-1.74	0.019	0.221
KHETCHOUMIAN_TRIM24_TARGETS_UP	-1.74	0.032	0.222
AMIT_EGF_RESPONSE_40_MCF10A	-1.74	0.023	0.222
JACKSON_DNMT1_TARGETS_DN	-1.73	0.015	0.222
HANN_RESISTANCE_TO_BCL2_INHIBITOR_DN	-1.73	0.004	0.222
BIOCARTA_EIF_PATHWAY	-1.73	0.020	0.223
JEON_SMAD6_TARGETS_UP	-1.73	0.032	0.223
SARRIO_EPITHELIAL_MESENCHYMAL_TRANSITION_DN	-1.73	0.008	0.223
DORN_ADENOVIRUS_INFECTION_12HR_UP	-1.73	0.008	0.223
PID_IL6_7_PATHWAY	-1.73	0.018	0.223
KANG_IMMORTALIZED_BY_TERT_UP	-1.73	0.002	0.224
YAMANAKA_GLIOBLASTOMA_SURVIVAL_UP	-1.73	0.004	0.224
PID_MYC_REPRESS_PATHWAY	-1.73	0.004	0.224
COATES_MACROPHAGE_M1_VS_M2_DN	-1.72	0.002	0.235
REACTOME_GROWTH_HORMONE_RECEPTOR_SIGNALING	-1.72	0.015	0.238
WINTER_HYPOXIA_UP	-1.72	0.030	0.238
REACTOME_GLUCURONIDATION	-1.72	0.020	0.238
SESTO_RESPONSE_TO_UV_C8	-1.72	0.002	0.239
DORSEY_GAB2_TARGETS	-1.72	0.025	0.241
LIANG_SILENCED_BY_METHYLATION_UP	-1.70	0.021	0.244
BIOCARTA_CDMAC_PATHWAY	-1.71	0.017	0.245
TSAI_DNAJB4_TARGETS_DN	-1.70	0.028	0.245
FARDIN_HYPOXIA_9	-1.70	0.019	0.245
TURJANSKI_MAPK7_TARGETS	-1.70	0.016	0.246
CAFFAREL_RESPONSE_TO_THC_UP	-1.70	0.026	0.246
JIANG_TIP30_TARGETS_UP	-1.71	0.008	0.246

Supplementary Table S3. *Cont.*

OLSSON_E2F3_TARGETS_DN	-1.70	0.027	0.246
GENTILE_UV_RESPONSE_CLUSTER_D2	-1.71	0.010	0.247
PID_AP1_PATHWAY	-1.70	0.008	0.247
ITO_PTTG1_TARGETS_UP	-1.70	0.026	0.247
BROWNE_HCMV_INFECTION_2HR_DN	-1.71	0.008	0.247
CAVARD_LIVER_CANCER_MALIGNANT_VS_BENIGN	-1.70	0.012	0.247
TENEDINI_MEGAKARYOCYTE_MARKERS	-1.71	0.006	0.247
GILDEA_METASTASIS	-1.70	0.026	0.247
MYLLYKANGAS_AMPLIFICATION_HOT_SPOT_7	-1.70	0.018	0.247
WOO_LIVER_CANCER_RECURRENCE_UP	-1.70	0.008	0.247
CASORELLI_ACUTE_PROMYELOCYTIC_LEUKEMIA_UP	-1.71	0.006	0.247
BURTON_ADIPOGENESIS_PEAK_AT_0HR	-1.70	0.006	0.247
WNT_SIGNALING	-1.70	0.010	0.247
SHAFFER_IRF4_TARGETS_IN_PLASMA_CELL_VS_MATURE_B_LYMPHOCYTE	-1.71	0.026	0.248
COLIN_PILOCYTIC_ASTROCYTOMA_VS_GLIOBLASTOMA_DN	-1.71	0.018	0.248
PID_S1P_S1P1_PATHWAY	-1.70	0.010	0.248
PID_VEGFR1_2_PATHWAY	-1.70	0.006	0.248
GROSS_HYPOXIA_VIA_HIF1A_DN	-1.70	0.016	0.248
CHIARADONNA_NEOPLASTIC_TRANSFORMATION_CDC25_UP	-1.71	0.010	0.248
GAJATE_RESPONSE_TO_TRABECTEDIN_UP	-1.69	0.020	0.249
WHITESIDE_CISPLATIN_RESISTANCE_DN	-1.70	0.026	0.249
GESERICK_TERT_TARGETS_DN	-1.71	0.029	0.249
REACTOME_REGULATION_OF_HYPOXIA_INDUCIBLE_FACTOR_HIF_BY_OXYGEN	-1.71	0.013	0.249

© 2019 by the authors. Licensee MDPI, Basel, Switzerland. This article is an open access article distributed under the terms and conditions of the Creative Commons Attribution (CC BY) license (http://creativecommons.org/licenses/by/4.0/).
